# Binary Cell Fate Decisions and Fate Transformation in the *Drosophila* Larval Eye

**DOI:** 10.1371/journal.pgen.1004027

**Published:** 2013-12-26

**Authors:** Abhishek Kumar Mishra, Maria Tsachaki, Jens Rister, June Ng, Arzu Celik, Simon G. Sprecher

**Affiliations:** 1 Institute of Cell and Developmental Biology, Department of Biology, University of Fribourg, Fribourg, Switzerland; 2 Center for Developmental Genetics, Department of Biology, New York University, New York, New York, United States of America; 3 Department of Molecular Biology and Genetics, Bogazici University, Bebek, Istanbul, Turkey; VIB and KU Leuven, Belgium

## Abstract

The functionality of sensory neurons is defined by the expression of specific sensory receptor genes. During the development of the *Drosophila* larval eye, photoreceptor neurons (PRs) make a binary choice to express either the blue-sensitive Rhodopsin 5 (Rh5) or the green-sensitive Rhodopsin 6 (Rh6). Later during metamorphosis, ecdysone signaling induces a cell fate and sensory receptor switch: Rh5-PRs are re-programmed to express Rh6 and become the eyelet, a small group of extraretinal PRs involved in circadian entrainment. However, the genetic and molecular mechanisms of how the binary cell fate decisions are made and switched remain poorly understood. We show that interplay of two transcription factors Senseless (Sens) and Hazy control cell fate decisions, terminal differentiation of the larval eye and its transformation into eyelet. During initial differentiation, a pulse of Sens expression in primary precursors regulates their differentiation into Rh5-PRs and repression of an alternative Rh6-cell fate. Later, during the transformation of the larval eye into the adult eyelet, Sens serves as an anti-apoptotic factor in Rh5-PRs, which helps in promoting survival of Rh5-PRs during metamorphosis and is subsequently required for Rh6 expression. Comparably, during PR differentiation Hazy functions in initiation and maintenance of *rhodopsin* expression. Hazy represses Sens specifically in the Rh6-PRs, allowing them to die during metamorphosis. Our findings show that the same transcription factors regulate diverse aspects of larval and adult PR development at different stages and in a context-dependent manner.

## Author Summary

Controlling cellular diversity requires a complex interplay of transcription factors. Using the *Drosophila* larval eye as genetic model we identify distinct mechanisms of how binary cell fate decisions are made, how sensory receptor gene expression is regulated and how cell fate identity is switched during metamorphosis. We show that the transcription factor Senseless fulfills three temporally and functionally separable roles in the same cells by (1) initiating a binary cell fate decision by controlling the cell fate determinants Spalt and Seven-up, (2) suppressing apoptosis during metamorphosis and (3) promoting Rhodopsin expression after metamorphosis. We further show that the transcription factor Hazy provides is required for early embryonic PR differentiation and that maintained Hazy expression is essential for Rhodopsin expression. Hazy provides a third function during metamorphosis by repressing Sens in one PR-subtype allowing it to undergo apoptotic cell death. We identified a novel mode of Rhodopsin regulation in which the highly conserved RCSI motif is dispensable for expression, demonstrating that the regulation of the Rhodopsin promoter is distinct in different visual organs. Our findings provide a unique example of how the same regulators control very distinct key aspects of development at distinct stages.

## Introduction

Even though the complexity of eyes varies between animal species, their function remains the same: perception of visual information from the environment. *Drosophila* employs simple eyes during the larval stage and complex compound eyes during adulthood. The adult compound eye is a widely used model system to study eye development, sensory receptor expression and function [1], [2]. However, only little is known regarding the development of the visual system in the larva. The larval eye (also termed Bolwig Organ, BO) is simple, but plays important roles in navigation, circadian rhythm and even the formation of associative memories [3], [4], [5], [6]. Each larval eye is composed of 12 photoreceptor neurons (PRs) that are divided into two subtypes depending on the *rhodopsin* gene they express. Four PRs express the blue-sensitive Rhodopsin 5 (Rh5), while the remaining eight PRs express the green-sensitive Rhodopsin 6 (Rh6) [7]. All PRs of the larval eye develop during embryogenesis and are fully functional at larval hatching [8]. The development of larval PRs occurs in a two-step process: first, three or four primary precursors are specified by expressing the proneural gene *atonal* (*ato*) [9], [10]. In a second step, primary precursors recruit surrounding cells to develop into secondary precursors through Epidermal Growth Factor Receptor (EGFR) signaling [9]. Subsequently, primary precursors differentiate into Rh5-PRs, while secondary precursors develop into Rh6-PRs. Interestingly approximately the same ratio of Rh5- to Rh6-expressing PRs (30∶70) exists in the adult retina [7], [11]. However, conversely to adult R8 PRs, where mutually exclusive expression of Rh5 and Rh6 is based on a stochastic mechanism [11], [12], [13], [14], Rh5 and Rh6 expression is initiated through a deterministic cell-fate specification mechanism in the larval eye.

Terminal differentiation and PR subtype specification in the larval eye requires the action of the transcription factors Spalt (Sal), Seven-up (Svp) and Orthodenticle (Otd) [7], three genes that are also involved in PR fate decision in the compound eye [15], [16], [17], [18]. During metamorphosis, the larval eye undergoes a transformation to become a group of extraretinal PRs (termed “eyelet”) involved in entrainment of the circadian clock [19], [20]. During this transformation, larval Rh5-PRs switch expression from Rh5 to Rh6, while Rh6-PRs undergo apoptotic cell death. Both processes are controlled by ecdysone signaling: interfering with Ecdysone Receptor (EcR) function in Rh6-PRs inhibits apoptosis, while inhibiting EcR signaling in Rh5-PRs blocks the switch of *rhodopsins* [21]. The genetic network acting downstream of EcR to control the *rhodopsin* switch or to induce apoptosis is currently unknown.

Here, we investigate the function of two key transcription factors controlling the development of the larval PRs and transformation of the larval eye to the adult eyelet. We show that the zinc finger transcription factor Senseless (Sens) acts in three steps in larval PR and eyelet development. First, a short pulse of Sens expression in primary precursors initiates a genetic feedforward loop to maintain the Rh5-cell fate, thereby acting as a binary switch between Rh5- versus Rh6-PR cell fates. Moreover, Sens provides a second function during metamorphosis to suppress EcR-induced apoptosis in the Rh5-PR subtype. Finally, Sens is also necessary to promote Rh6 expression in the adult eyelet.

We further show that the homeodomain transcription factor Hazy (Flybase: Pph13 for PvuII-PstI homology 13) has two distinct roles during larval eye development and a third one during metamorphosis. Hazy is necessary for the initiation and maintenance of Rh5 and Rh6 expression in the larval eye, while initial subtype specification occurs normally. Hazy acts through a conserved motif present in the *rhodopsin* promoters, called Rhodopsin Core Sequence I (RCSI) [22], [23], [24]. The analysis of RCSI function led to two new findings that differ from the situation in the adult retina: First, Hazy acts through the RCSI of both Rh5 and Rh6 in the larva, whereas it affects only Rh6 in the adult. Second, neither the RCSI nor Hazy are required for activation of Rh6 in the eyelet, demonstrating that the regulation of the *rh6* promoter is distinct in the larval and adult eyes compared to the adult eyelet. During metamorphosis, Hazy represses Sens in Rh6-PRs, thus allowing them to undergo apoptosis. Our findings show that a small set of transcription factors are used to regulate diverse aspects of larval and adult PR development at different stages and in a context-dependent manner.

## Results

### An early pulse of Sens initiates a feedforward loop to maintain primary precursor differentiation into Rh5-PRs

Specification of larval Rh5-PRs depends on the combinatorial action of Sal and Otd [7]. Sal is exclusively expressed in Rh5-PRs and promotes their differentiation. In *sal* mutants, primary precursors fail to fully differentiate and remain “empty” PRs lacking *rhodopsin* expression. Otd promotes Rh5 and represses Rh6 expression in Rh5-PRs. Conversely, Svp is exclusively expressed in Rh6-PRs, where it represses Sal and promotes Rh6 expression [7]. However, the mechanisms of how the cell fate choice of primary and secondary precursors is initially controlled remain unknown.

**Figure 1:**
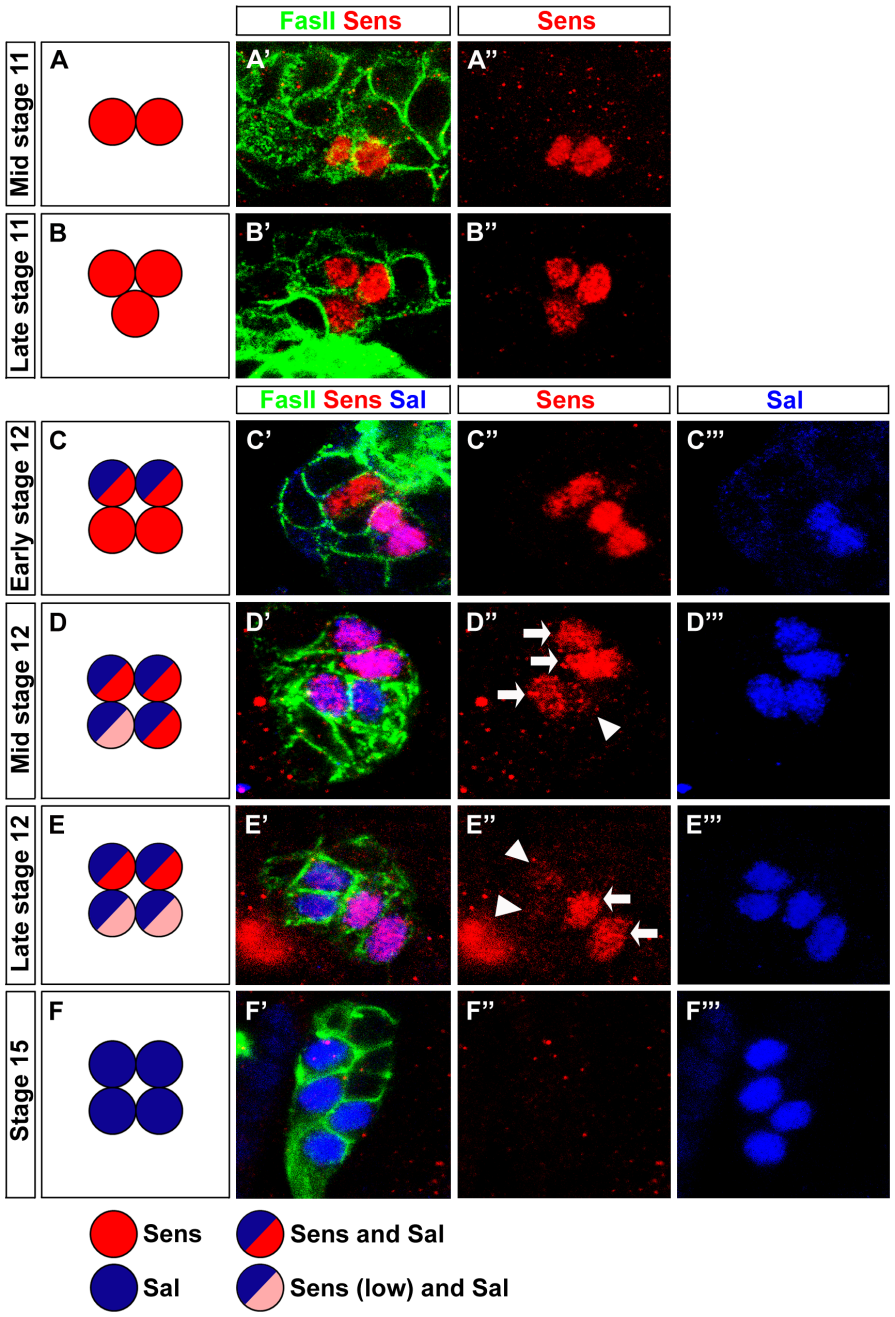
Pulsed Sens expression during precursor development. (A–F) Schematic representation of Sens and Sal expression during stage 11 and stage 12 in wild-type embryonic PRs. (A′–F′) Sens expression (red) from stage 11 to 15 in wild-type embryonic PRs stained with anti-FasII (green) and anti-Sal (blue); single confocal sections are shown. (A′, A″) Sens staining in mid stage 11 is detected in two cells. (B′, B″) In late stage 11, Sens staining is detected in three cells. (C′, C″, C‴) Sens staining in early stage 12 in four cells, co-expressed with Sal in two cells. (D′, D″, D‴) At mid stage 12, all cells express Sal, co-expression with Sens is found in three cells (high, arrow) and one cell (low, arrowhead). (E′, E″, E‴) Sens staining in late stage 12, all cells express Sal, co-expression with Sens is restricted to two cells (high) and very low residual expression is detected in the remaining two cells. (F′, F″, F‴) No Sens expression is seen at stage 15, while Sal expression is observed in all four cells.

**Figure 2:**
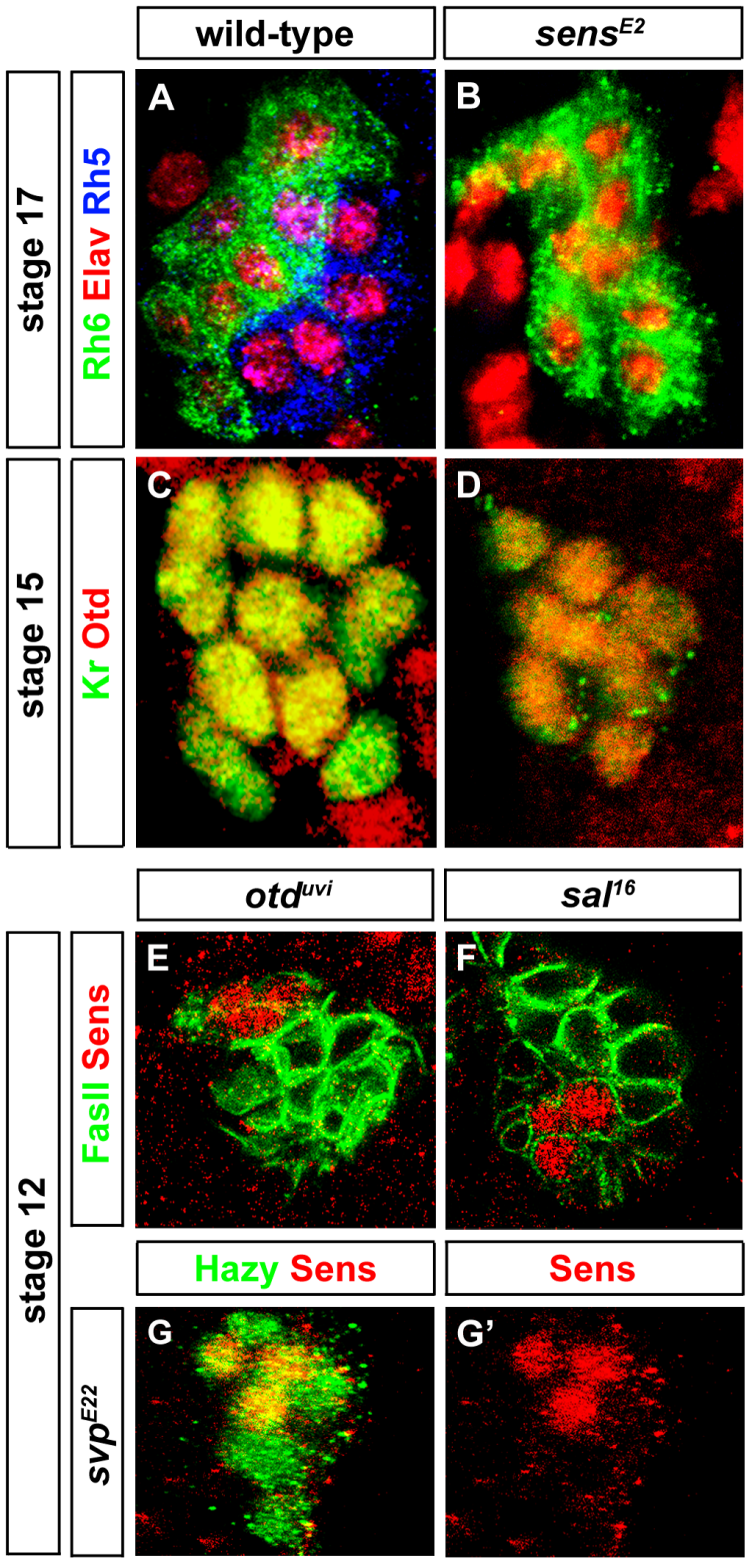
Sens is required for Rh5-PR identity and acts in parallel with Otd. (A, B) Rh5 and Rh6 expression in wild-type and *sens^E2^* mutant PRs during embryonic stage 17, stained with anti-Rh6 (green), anti-Rh5 (blue) and anti-Elav (red); z-projection of confocal sections. No Rh5 expression was seen in *sens^E2^* mutants and all the cells were marked by Rh6. (C, D) Otd expression in wild-type and *sens^E2^* mutant PRs during embryonic stage 15 stained with anti-Kr (green) and anti-Otd (red). Both in wild-type and *sens^E2^* mutants, all the PR nuclei expresses Otd, showing that Otd was not affected in *sens^E2^* mutant; z-projection of confocal sections. (E, F, G) Sens expression (red) in *otd^uvi^*, *sal^16^* and *svp^E22^* mutant PRs during embryonic stage 12. Staining against FasII or Hazy (green), shows that Sens expression was not affected in these mutants; z-projection of confocal sections.

Since Sens is involved in initial specification steps in PRs of the adult compound eye [25], [26], we tested whether it has a similar role in the larval eye. First, we analyzed the expression of Sens. During embryonic stage 11, when primary and secondary precursors have been formed, cells of the larval eye detach from the optic lobe placode and start to express differentiation markers such as, Elav, Fasciclin II (FasII), Krüppel (Kr) and Hazy [27] (see below). Sens is specifically expressed in all primary precursors in a short highly dynamic pulse during embryonic stages 11 and 12 (Figure 1A–1E). Sens expression initiated first in two primary precursors at mid stage 11, and is subsequently upregulated in the remaining two primary precursors (Figure 1A, 1B and 1C). Expression of Sens ceases during mid-late stage 12, when primary precursors start to express Sal (Figure 1C, 1D and 1E), which is then maintained until their maturation into fully differentiated Rh5-PRs (Figure 1C, 1D, 1E and 1F). Since Sens is exclusively and transiently expressed in the precursors of the Rh5-PR subtype, we analyzed the expression of Rh5 and Rh6 in *sens* mutants at the end of embryogenesis when these mutants die. Even though the correct number of PRs is produced, no Rh5 expression can be found, while all 12 PRs express Rh6 (Figure 2B).

Since *sens* mutants display the same *rhodopsin* expression phenotype as *otd* mutants [7], i.e., loss of Rh5 and gain of Rh6, we investigated the interactions between *otd* and *sens*. In *sens* mutants, all PRs express Otd comparable to wild-type (Figure 2C and 2D) and therefore, Otd does not act downstream of Sens. Conversely, we tested if *sens* expression depends on Otd. No change of Sens expression is observed in *otd* mutants (Figure 2E), suggesting that Sens and Otd act in parallel.

**Figure 3:**
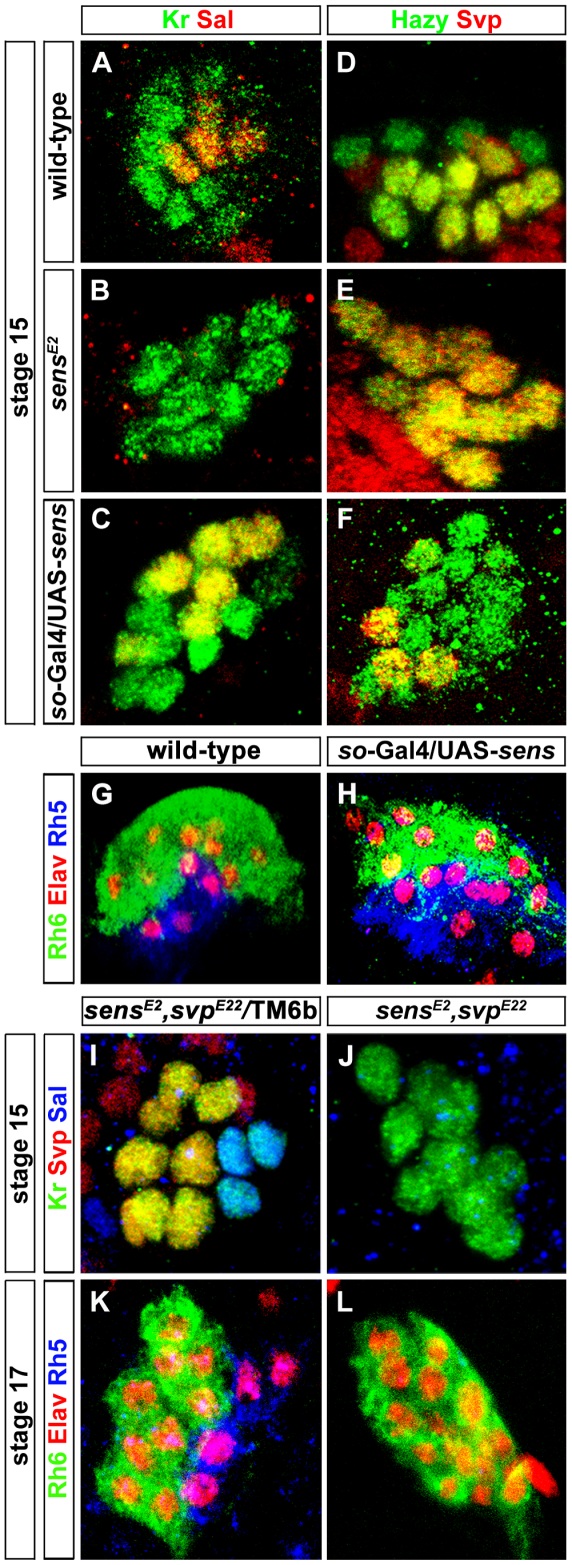
Role of Sens in regulation of precursor differentiation and *rhodopsin* expression. (A, B, C) Sal expression (red) in wild-type, *sens^E2^* mutant and Sens over-expression (*so*-Gal4/UAS-*sens*) PRs during embryonic stage 15 stained with anti-Kr (green); z-projection of confocal sections. Sal expression was not detected in the *sens^E2^* mutant, while an increase of Sal expressing cells was found in *so*-Gal4/UAS-*sens* over-expression, showing that Sens genetically interacts with Sal and promotes its activation (D, E, F) Svp expression (red) in wild-type, *sens^E2^* mutant and Sens over-expression (*so*-Gal4/UAS-*sens*) PRs during embryonic stage 15, stained with anti-Hazy (green); z-projection of confocal sections. Svp was de-repressed in *sens^E2^* mutant in all PRs, while a reduced number of Svp expressing cells was found in *so*-Gal4/UAS-*sens* over-expression, suggesting that Sens genetically interacts with Svp and promotes its repression. (G, H) Rh5 (blue) and Rh6 (green) expression in wild-type and *so*-Gal4/UAS-*sens* over-expression in third instar larval eye, stained with anti-Elav (red); z-projection of confocal sections. An increased number of Rh5 expressing cells were found in *so*-Gal4/UAS-*sens* over-expression. (I, J) Sal expression (Blue) in the heterozygous (control) and homozygous (*sens^E2^*, *svp^E22^*) double mutant PRs during stage 15, stained with anti-Kr (green) and anti-Svp (red); z-projection of confocal sections. Sal expression was not found in the homozygous double mutant PRs. (K, L) Rh5 and Rh6 expression in heterozygous (control) and homozygous (*sens^E2^*, *svp^E22^*) double mutant PRs during stage 17, stained with anti-Rh6 (green), anti-Elav (red) and anti-Rh5 (blue); z-projection of confocal sections. Rh5 expression was absent in the homozygous double mutant PRs, whereas all PRs expressed Rh6.

**Figure 4:**
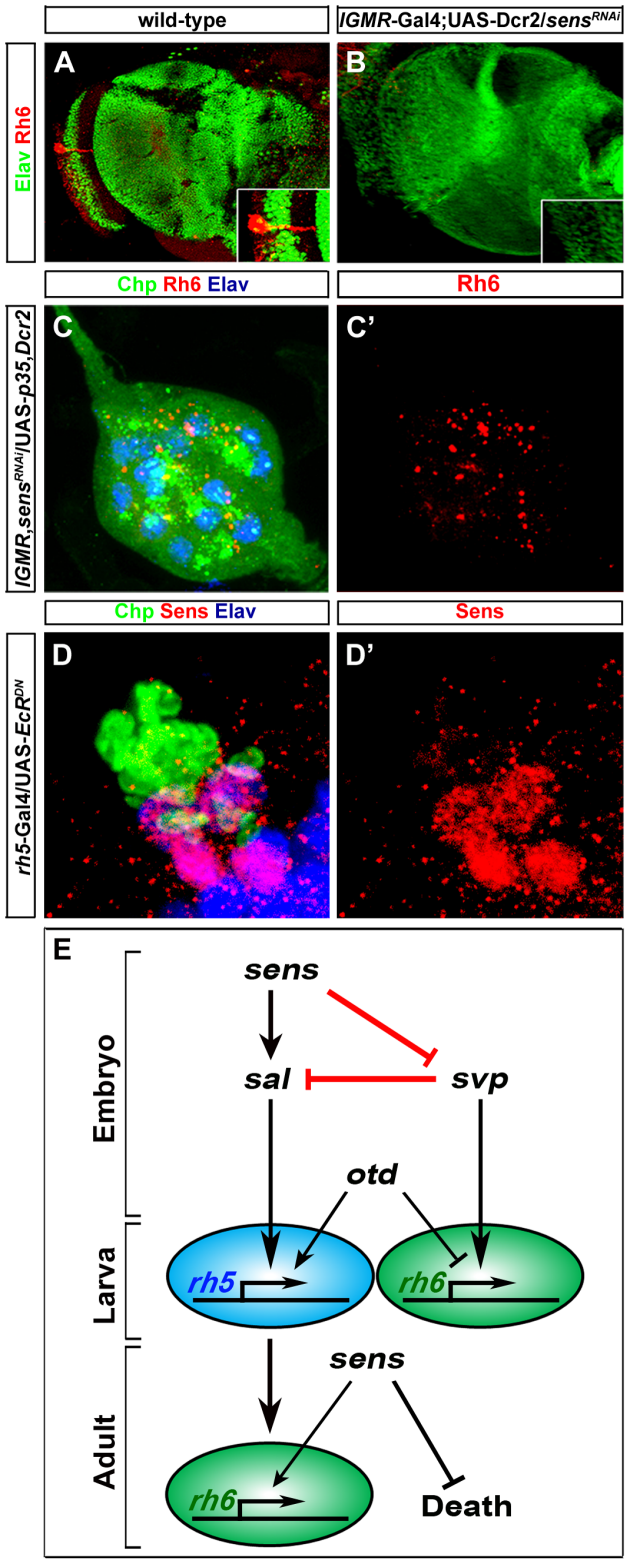
Role of Sens in the transformation of larval eye into the adult eyelet. (A, B) Rh6 expression in wild-type and *sens^RNAi^* (*lGMR*-Gal4; UAS-*Dcr2*/UAS-*sens^RNAi^*) adult eyelets, stained with anti-Rh6 (red) and anti-Elav (green). In all *lGMR*-Gal4; UAS-*Dcr2*/UAS-*sens^RNAi^* animals, the eyelet was absent (inset: high magnification of eyelet position). (C, C′) Rh6 expression (red) in *sens^RNAi^* when *p35* was ectopically expressed in the eyelet to keep the cells alive (UAS-*sens^RNAi^*/*lGMR*-Gal4; UAS-*p35*/UAS-*Dcr2*), stained with anti-Chp (green), and anti-elav (blue); z-projection of confocal sections. No Rh6 expression was found in the eyelet. (D, D′) Sens expression (red) in the eyelet when a dominant-negative form of EcR was ectopically expressed in Rh5-PRs (*rh5*-Gal4/UAS-*EcR^DN^*) and stained with anti-Chp (green) and anti-Elav (blue); z-projection of confocal sections. Sens was expressed in all four eyelet cells. (E) A model describing the role of Sens during different developmental stages.

Since both Sal and Svp are key factors orchestrating differentiation of Rh5- and Rh6-PRs, we analyzed the interaction between *sens* and *sal* or *svp*. In primary precursors, the pulse of Sens precedes Sal expression, suggesting that Sens regulates Sal. Indeed, in *sens* mutants, Sal expression is abolished (Figure 3B). Conversely, no change of Sens expression is observed in *sal* mutants (Figure 2F), suggesting that Sens acts genetically upstream of *sal* and its transient expression may function as a trigger that initiates Sal expression. Moreover, in *sens* mutants, all precursors express Svp, the repressor of Rh5-PR fate (Figure 3E), but Sens expression is normal in *svp* mutants (Figure 2G and 2G′). Thus, Sens acts upstream of both Sal and Svp: it has a dual role in primary precursor cell fate specification, as it a) genetically promotes the expression of Sal (an activator of Rh5 fate) and b) genetically represses Svp (a repressor of *sal* and Rh5 fate) (Figure 4E).

Since Sens regulates Svp and Sal in primary precursors, we next addressed whether Sens is sufficient for genetically activating *sal* and repressing *svp* in precursors of the larval eye. We ectopically expressed *sens* under the control of *sine oculis*-Gal4 (*so*-Gal4), which starts to be expressed early in precursors of the optic epithelium. Ectopic expression of Sens results in an increased number of Sal expressing cells and a reduction of Svp expressing cells (Figure 3C and 3F). This is in line with the data above that Sens acts to promote *sal* and to inhibit *svp* expression. The switch of Svp-expressing precursors to Sal-expressing precursors suggests that some of the secondary precursors have changed their identity to primary precursors, and thus, might have switched their Rhodopsin expression. Indeed, we found an increase of Rh5-PRs and a decrease of Rh6-PRs, while the overall number of PRs remained unaltered in *so*-Gal4/UAS-*sens* larvae (Figure 3H). Our findings support a model in which a pulsed expression of Sens acts to allow primary precursors to adopt Rh5-cell fate by genetically repressing the default Rh6-cell fate (Figure 4E).

*svp* is not only necessary for Rh6 expression, but also for the repression of *sal* in secondary precursors [7]. We therefore next investigated whether Sens-dependent Sal expression in primary precursors occur in a Svp-dependent or independent manner. In other words, Sal expression could be either due to direct activation by Sens or it could be an indirect result of Sens repressing Svp, which in turn represses Sal. To address this, we generated a *sens*, *svp* double mutant. If the activation of Sal is an indirect consequence of Svp repression by Sens, we would expect to observe a de-repression of Sal in all the PRs. Conversely if it is Svp-independent, we would expect to see the same phenotype as in *sens* mutant alone. We found that in *sens, svp* double mutants, Sal expression was still absent (Figure 3J), further suggesting that Sal expression is most likely due to activation by *sens* and not indirect due to relief or repression by Svp.

We next investigated whether Sens also functions genetically downstream of the Sal/Svp fate decision to regulate Rh5 expression or whether ectopic *sal* expression or loss of *svp* in *sens* mutants can rescue Rh5 expression. To address this, we investigated Rh5 and Rh6 expression in *sens*, *svp* double mutants at the end of embryogenesis, when these mutants die. Although the same number of PRs was produced compared to the wild-type, no Rh5 expression was found and all of them expressed Rh6 (Figure 3K and 3L). This finding suggests that Sens does not function downstream of Svp to regulate Rh5 expression.

We have previously shown that EGFR signaling is required to inhibit apoptosis in secondary precursors during the formation of the larval eye. Genetically inhibiting the EGFR pathway results in a larval eye comprising only 3–4 Rh5-expressing PRs [7], [9]. We next tested if EGFR signaling is also required for Sens expression in the developing primary precursors. We found that Sens expression was normal compared to the wild-type in the remaining primary precursors when inhibiting EGFR signaling by ectopic expression of a dominant negative form of EGFR (UAS-*EGFR^DN^*) under the control of *so*-Gal4 (Figure S1A and S1B). Thus, expression of Sens in developing PRs during embryogenesis occurs independently of EGFR signaling. *ato* has been shown to promote Sens expression in the eye antennal disc [25]. Since in *ato* mutant embryos primary precursors fail to develop, we were unable to assess if *ato* is necessary to promote Sens expression. Instead, we tested if ectopic expression of *ato* in all precursors is sufficient to induce and maintain Sens expression in the larval eye. However, ectopic expression of *ato* was not sufficient to induce Sens expression during embryogenesis (Figure S1E and S1F).

### Reinitiated Sens expression in Rh5-PRs is required for cell survival during metamorphosis and Rh6 expression in the adult eyelet

During late third instar larval stage, Sens expression is reinitiated in Rh5-PRs and is maintained during metamorphosis, when these cells transform to become the adult eyelet [21]. To address the role of Sens in Rh5-PRs at this stage, we knocked-down Sens in all larval PRs by expressing *sens^RNAi^* together with *Dicer-2* (*Dcr-2*), which has been shown to enhance RNAi potency [28] with the pan-PR *lGMR*-Gal4 driver. *sens* knockdown leads to a complete loss of eyelet PRs (Figure 4B). Rh6-PRs, which do not express Sens, undergo normal EcR mediated apoptotic cell death [21]. Misexpression of Sens in these cells is sufficient to inhibit apoptosis [21]. Thus, Sens acts as a PR-subtype specific survival factor for Rh5-PRs that become the adult eyelet (Figure 4E).

We next addressed whether besides blocking apoptotic cell death Sens might also be required for *rhodopsin* expression in the eyelet. We therefore knocked down *sens* in all larval PRs, but at the same time we kept the PRs alive by concomitantly expressing the apoptosis inhibitor *p35* with the *lGMR*-Gal4 driver. This resulted in eyelets consisting of 12 PRs that failed to express Rh6 expression (Figure 4C and 4C′), suggesting that Sens is also required for Rh6 expression in the eyelet (Figure 4E).

Ecdysone signaling is required for the transformation of the larval eye into the adult eyelet. Genetically inhibiting EcR signaling in Rh6-PRs blocks apoptosis, while inhibiting EcR signaling in Rh5-PRs blocks the switch of *rhodopsins* [21]. We next asked if EcR signaling is required for Sens expression in the adult eyelet by ectopically expressing a dominant negative form of EcR (UAS-*EcR^DN^*) in Rh5-PRs using *rh5*-Gal4. Sens expression was unaltered (Figure 4D and D′), indicating that Sens expression in the eyelet is independent of EcR signaling.

In summary, Sens fulfills three temporally and functionally separable roles in the same cells at different developmental stages: First, it initiates precursor specification in early embryonic stages; second, it suppresses apoptosis and thus enables survival during metamorphosis in fully differentiated Rh5-PRs; and third, it is required in the eyelet for Rh6 expression (Figure 4E).

### Hazy is not required for larval PR subtype specification

**Figure 5:**
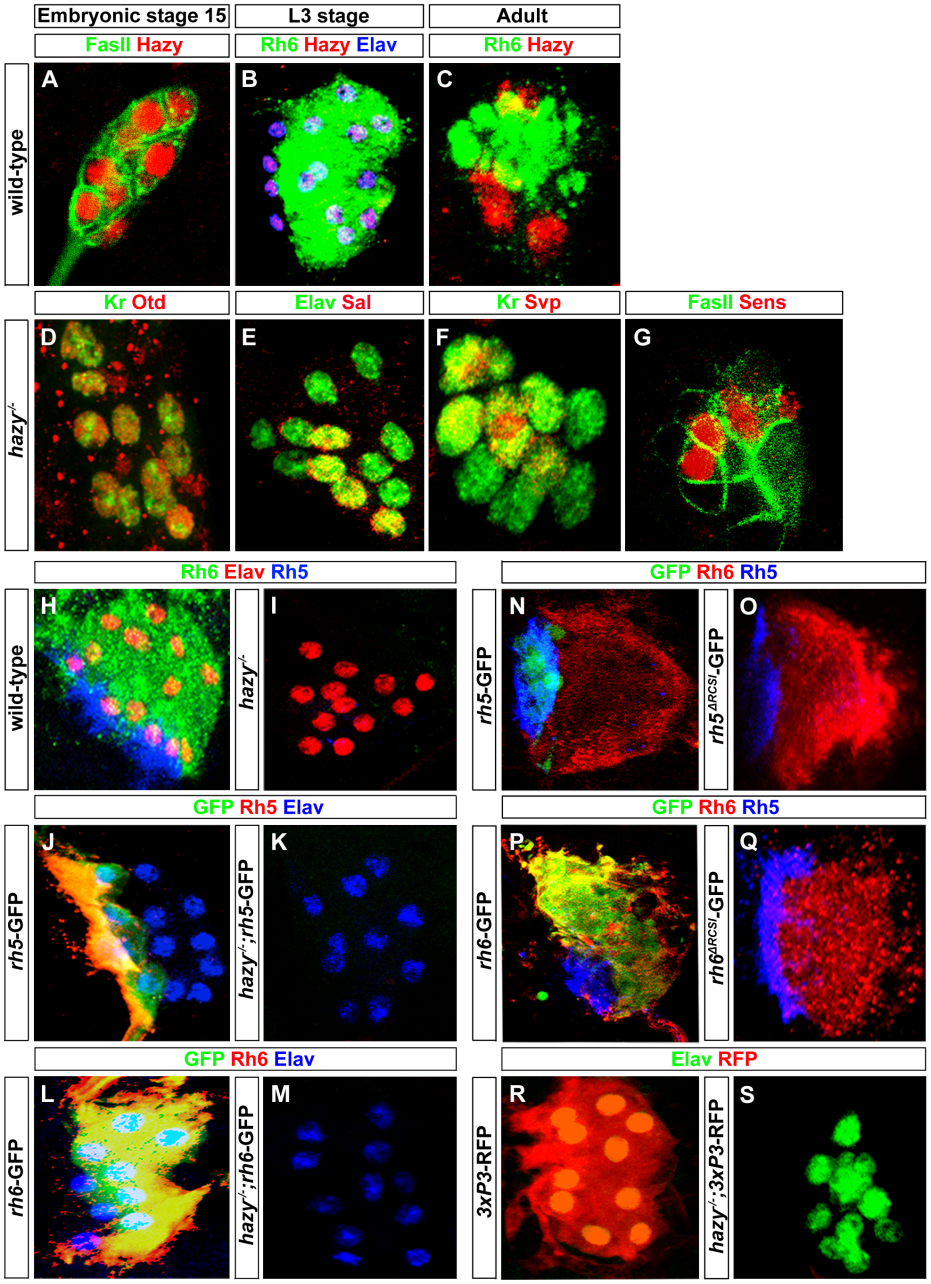
Hazy functions in larval eye development by acting through the promoters of *rh5* and *rh6*. (A) Wild-type embryonic larval eye precursors of stage 15 stained with anti-Hazy (red) and anti-FasII (green); single confocal section. (B) Wild-type third instar larval eye stained with anti-Hazy (red), anti-Rh6 (green) and anti-Elav (blue). (C) Wild-type adult eyelet stained with anti-Hazy (red) and anti-Rh6 (green); z-projection of confocal sections. Hazy was expressed in all the PRs of embryonic larval eye precursors, third instar larval eye and in adult eyelet. *hazy^−/−^* mutant larval eyes stained with (D) anti-Otd (red) and anti-Kr (green), (E) anti-Elav (green) and anti-Sal (red). Embryonic larval eye precursors were stained with (F) anti-Kr (green) and anti-Svp (red), (G) anti-FasII (green) and anti-Sens (red). No change of Otd, Kr, Sal, Svp, and Sens expression was observed in *hazy^−/−^* mutants. (H, I) Wild-type and *hazy^−/−^* mutant third instar larval eyes stained with anti-Elav (red), anti-Rh6 (green) and anti-Rh5 (blue). Rh5 and Rh6 expression was lost in *hazy^−/−^* mutants. (J, K) Third instar larval eyes of wild-type reporter line of *rh5* (*rh5*-GFP) and *rh5*-GFP in *hazy^−/−^* null background (*hazy^−/−^*; *rh5*-GFP), stained with anti-Rh5 (red), anti-GFP (green) and anti-Elav (blue). (L, M) Third instar larvae of wild-type reporter line of *rh6* (*rh6*-GFP) and *rh6*-GFP in *hazy^−/−^* null background (*hazy^−/−^*; *rh6*-GFP) stained with anti-Rh6 (red), anti-GFP (green) and anti-Elav (blue); z-projection of confocal sections. No GFP expression was observed in the *hazy^−/−^* mutant background. (N, O, P, Q) *rh5*-GFP, *rh5^ΔRCSI^*-GFP, *rh6*-GFP and *rh6^ΔRCSI^*-GFP larval eyes stained with anti-Rh6 (red), anti-GFP (green) and anti-Rh5 (blue); z-projection of confocal sections. No GFP expression was observed in the larval eye of *rh5^ΔRCSI^*-GFP (O) and *rh6^ΔRCSI^*-GFP animals (Q). (R, S) Third instar larval eyes of *3XP3*-RFP and *3XP3*-RFP in *hazy^−/−^* null background (*hazy^−/−^*; *3XP3*-RFP), stained with anti-RFP (red) and anti-Elav (green). No RFP expression was observed in the larval eye in *hazy^−/−^*; *3xP3*-RFP animals.

Hazy is expressed in developing precursors of the larval eye [27] and we therefore investigated its function in larval PRs. Hazy is expressed in all larval PRs starting in precursors at embryonic stage 12 (Figure 2G) and continues to be expressed throughout embryogenesis (Figure 5A). Hazy expression is further maintained in fully differentiated larval PRs (Figure 5B) and PRs of the adult eyelet (Figure 5C). Since Hazy is already expressed early in both precursor types, we investigated initial specification of primary and secondary precursors in *hazy* mutants. Specification of precursors appears to occur normally since Otd, Sal, Svp and Sens expression was not affected (Figure 5D, 5E, 5F and 5G). In *hazy* mutants, PRs differentiate normally as indicated by the expression of canonical PR differentiation markers such as Kr, Elav or FasII (Figure 5D, 5E and 5G). We next addressed if EGFR signaling is required for Hazy expression in PR precursors. We found that Hazy expression was not altered when blocking EGFR signaling (Figure S1C and S1D).

### Hazy controls *rhodopsin* expression in the larval eye

Since Hazy controls *rh6* expression, but not *rh5* expression in the adult retina [29], we next investigated expression of Rh5 and Rh6 in *hazy* null mutant larvae. Neither Rh5 nor Rh6 expression was detected (Figure 5I). To address whether the lack of *rh5* and *rh6* expression occurs at the transcriptional level, we used *rh5*-GFP and *rh6*-GFP reporter lines: GFP expression was completely abolished in both *hazy; rh5*-GFP and *hazy; rh6*-GFP mutants (Figure 5J, 5K, 5L and 5M). Thus, Hazy is necessary for expression of both Rhodopsins in the larval eye, whereas it is only required for Rh6 in the adult eye.

In the adult retina, it has been suggested that Hazy acts through the *rhodopsin* core sequence I (RCSI) that is found in all proximal *rhodopsin* promoters [23], [30], [31]. We used *rh5^ΔRCSI^ -GFP* and *rh6^ΔRCSI^ -GFP* reporter lines in which the RCSI is deleted to study the requirement of the RCSI in the larval eye. In both cases, we observed a complete loss of GFP expression (Figure 5N, 5O, 5P and 5Q), which demonstrates that the RCSI is necessary for *rh5* and *rh6* expression in larval PRs. Moreover, Hazy is required for the activation of a generic version of the RCSI called P3 that is sufficient when multimerized to drive reporter expression in all PRs [30]. After introducing the 3×P3-RFP in a *hazy* mutant background, we observed a complete lack of RFP expression in the larval eye (Figure 5R and 5S). This result further supports that Hazy acts directly through the RCSI sites of *rh5* and *rh6* to activate their expression in larval PRs.

### Temporal rescue unveils a dual role of *hazy* in Rhodopsin expression

**Figure 6:**
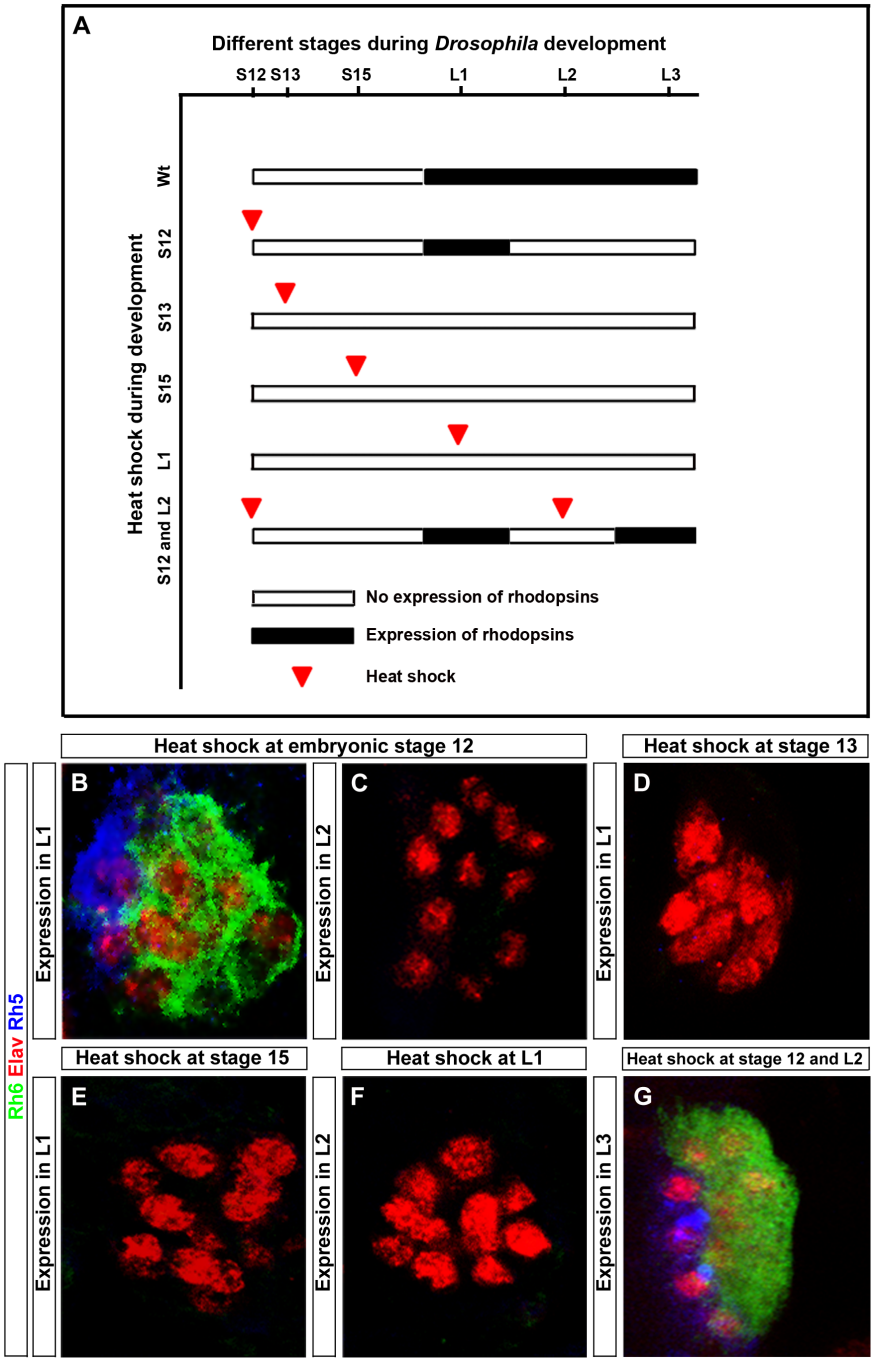
Rescue of the *hazy^−/−^* mutant phenotype in the larval eye. (A) Heat shock mediated rescue of *hazy^−/−^* mutant at different stages during development and consequence for Rhodopsin expression. White bar indicates no Rhodopsin expression, while black bar indicates Rhodopsin expression. Red arrowhead marks the stage at which heat shock was given. (B–G) All panels show larval eyes stained with anti-Elav (red), anti-Rh6 (green) and anti-Rh5 (blue); z-projection of confocal sections. (B, C) Heat shocks were performed three times at embryonic stage 12. At the L1 stage, Rh5 and Rh6 expression was detected (B), while at L2 stage, Rh5 and Rh6 expression was lost (C). Heat shocks performed at stage 13 (D), stage 15 (E) and at L1 stage (F) did not result in a rescue of Rh5 and Rh6 expression in the larval eyes at L1 in (D, E) and L2 in (F). Heat shocks at embryonic stage 12 and at L2 stage restores Rh5 and Rh6 expression in L3 larvae (G).

In order to address at which time point during PR development Hazy functions, we rescued the *hazy* mutant phenotype by expressing *hazy* under the control of a heat shock inducible promoter (hs-*hazy*) at distinct developmental stages. A heat-shock was given at 37°C for 30 minutes and *rhodopsin* expression was assessed after larval hatching. Heat shocks at embryonic stage 12 resulted in a rescue of both Rh5 and Rh6 expression in the first larval instar (Figure 6B). However, the expression of *rhodopsins* was not maintained: We neither detect Rh5 nor Rh6 in the second larval instar (Figure 6C). This suggests that Hazy expression is continuously required to maintain Rhodopsin expression. To further test this, we took animals that had received a heat shock at embryonic stage 12 and applied a second heat shock during the second larval instar. Indeed, Rh5 and Rh6 expression was restored in the third larval instar (Figure 6G), supporting that continuous Hazy expression is essential for maintained Rh5 and Rh6 expression.

Surprisingly, heat shocks after stage 12 (during embryonic stage 13, 15 or even in the first instar) did not rescue the lack of *rhodopsin* expression (Figure 6D, 6E and 6F). Since *rhodopsin* expression starts at embryonic stage 16/17, Hazy appears to provide an important function at embryonic stage 12 during the specification process from precursors to PRs prior to its role in *rhodopsin* regulation.

Thus, Hazy is playing two distinct roles in the larval eye: first, it is required during embryogenesis for proper PRs differentiation, and second, expression of Hazy throughout development is essential for larval PRs to express Rh5 and Rh6.

### Hazy is necessary for apoptosis of larval Rh6-PRs

**Figure 7:**
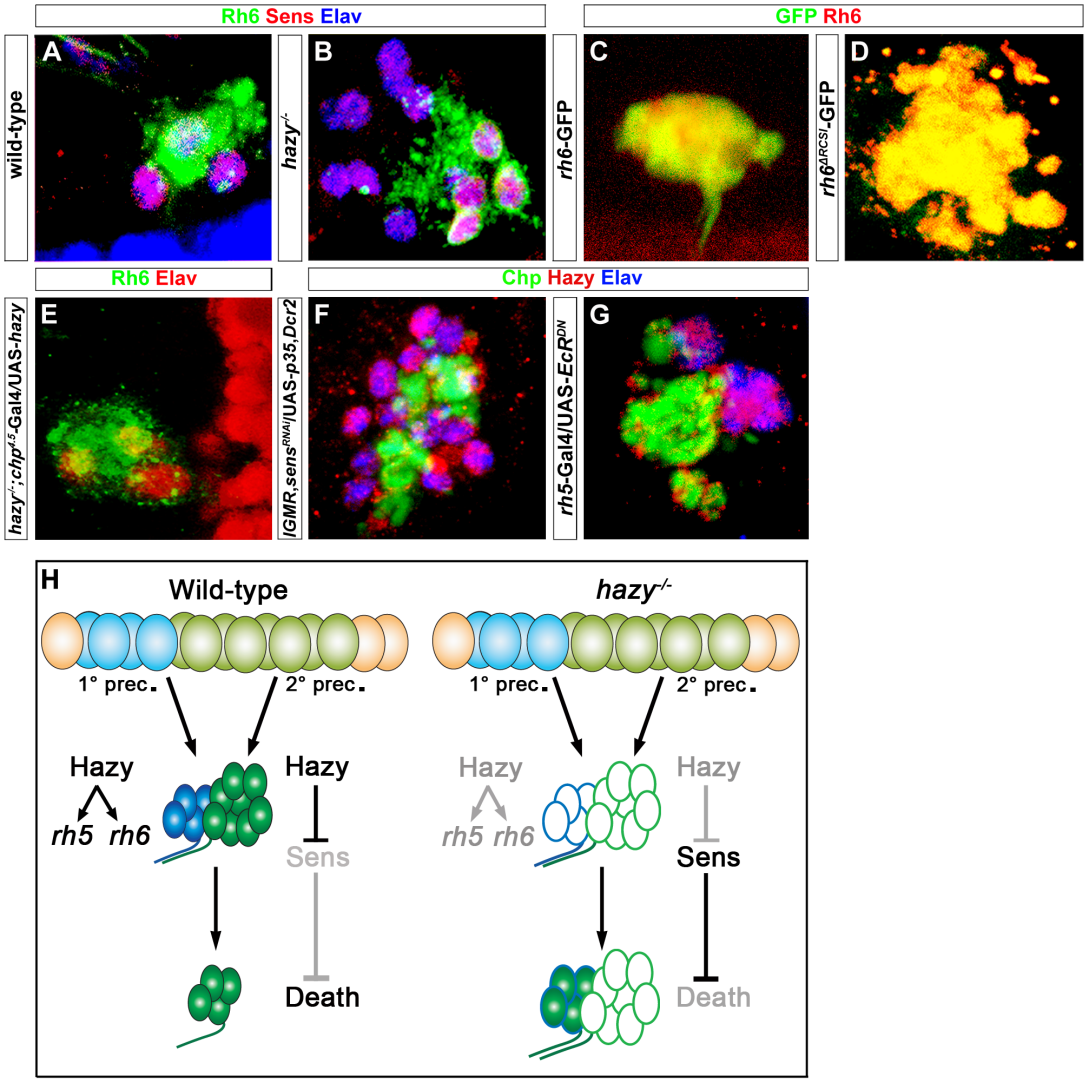
Role of Hazy in the adult eyelet. (A, B) Wild-type and *hazy^−/−^* mutant eyelets were stained against Sens (red), Rh6 (green) and Elav (blue); z-projection of confocal sections. In *hazy^−/−^* mutant, the eyelet consisted of 12 cells and all of them expressed Sens, while Rh6 expression was restricted to four cells. (C, D) *rh6*-GFP and *rh6^ΔRCSI^*-GFP eyelets, stained with anti-Rh6 (red) and anti-GFP (green); z-projection of confocal sections. GFP expression was still observed in the eyelets of *rh6^ΔRCSI^*-GFP. (E) UAS-*hazy* was expressed under the control of *chp^4.5^*-Gal4 in a *hazy^−/−^* null background (*hazy^−/−^; chp^4.5^*-Gal4/UAS-*hazy*) and the adult eyelet was analyzed for Elav (red) and Rh6 (green); z-projection of confocal sections. Normal number of Rh6 expressing PRs was found in *hazy^−/−^; chp^4.5^*-Gal4/UAS-*hazy* animals. (F) Hazy expression was assessed in *sens^RNAi^* when *p35* was ectopically expressed in the eyelet to keep the cells alive (UAS-*sens^RNAi^*/*lGMR*-Gal4; UAS-*p35*/UAS-*Dcr2*) and stained with anti-Hazy (red), anti-Chp (green) and anti-Elav (blue). Eyelet consists of 12 cells and Hazy was expressed in all the cells in UAS-*sens^RNAi^*/*lGMR*-Gal4;UAS-*p35*/UAS-*Dcr2* animals; z-projection of confocal sections. (G) Hazy expression (red) when a dominant-negative form of EcR was ectopically expressed in Rh5-PRs (*rh5*-Gal4/UAS-*EcR^DN^*) in the eyelet and stained with anti-Chp (green) and anti-Elav (blue); z-projection of confocal sections. Eyelet consists of four cells and Hazy was expressed in all PR cells. (H) A Model describing the role of Hazy in the larval eye and the adult eyelet.

Hazy expression is maintained in larval PRs throughout metamorphosis to the adult eyelet (Figure 5C). We therefore analyzed *rhodopsin* expression in *hazy* mutant eyelets. Surprisingly, we found that originally “empty” Rh5-PRs correctly turn on *rh6* during metamorphosis (Figure 7B). Thus, in the adult eyelet, Rh6 expression depends on Sens (see above), but occurs independently of Hazy, while in the larval eye as well as in the adult retina, Hazy is essential for Rh6 expression. As Hazy is not required for Rh6 expression in the eyelet, we reasoned that the RCSI should be dispensable for activation of the *rh6*-GFP reporter. Indeed, GFP expression was still observed in the eyelet when the RCSI was deleted from *rh6*-GFP (Figure 7D), further supporting that *rh6* regulation is distinct in the larval eye and the adult eye from the eyelet.

Interestingly, in *hazy* mutants, larval Rh6-PRs do not undergo apoptosis during pupation and are maintained into the adult, leading to a bigger eyelet that consists of about 12 PRs (Figure 7B). The larval Rh5-PRs switch on Rh6 expression during metamorphosis, while the former larval Rh6-PRs are empty. Since we have identified Sens as a survival factor counteracting EcR-induced apoptosis (see above), we investigated if Sens is expressed in the adult eyelet in *hazy* mutants and we found that this is indeed the case (Figure 7B). Therefore, Hazy is necessary to repress Sens in Rh6-PRs to allow them to die during metamorphosis. In line with this conclusion, the mutant phenotypes could be rescued with a PR-specific *chaoptin*-Gal4 driver (*chp^4.5^*-Gal4), driving *hazy* (Figure 7E; Figure S2).

We next assessed the genetic interaction between Hazy and Sens in the eyelet. Genetic knock-down of *sens* using RNAi, while inhibiting apoptosis by expressing *p35* results in an eyelet consisting of 12 Hazy expressing PRs (Figure 7F), supporting that Hazy expression does not depend on Sens.

We next investigated if Hazy expression in the eyelet depends on EcR signaling. We expressed EcR^DN^ in Rh5-PRs using *rh5*-Gal4 and did not observe a change in Hazy expression (Figure 7G), suggesting that Hazy expression in the eyelet is independent of EcR signaling.

Taken together, these results provide evidence for distinct functions of Hazy for larval PR development and their transformation into the adult eyelet: First, Hazy is necessary for the differentiation of PRs during embryogenesis; second, maintained Hazy expression promotes Rh5 and Rh6 expression in larval PRs; third, in Rh6-PRs Hazy is necessary to repress *sens* during metamorphosis, allowing these cells to undergo apoptotic cell death (Figure 7H).

## Discussion

### Sens initiates a binary cell fate decision in larval PRs

In the larval eye, determination of primary or secondary precursors to acquire either Rh5-PR or Rh6-PR identity depends on the transcription factors Sal, Svp and Otd [7]. Primary as well as secondary precursors have the developmental potential to express Rh5 or Rh6. During differentiation, a pulsed expression of Sens acts as a trigger to initiate a distinct developmental program: Sens acts genetically in a feedforward loop to inhibit the Rh6-PR cell-fate determinant Svp and to promote the Rh5-PR cell-fate determinant Sal. Similarly, in the adult retina, differentiation of ‘inner’ PRs R7 and R8 requires *sens* and *sal* [32], [33]. Sal is necessary for Sens expression in R8-PRs and misexpression of Sal is sufficient to induce Sens expression in the ‘outer’ PRs R1-R6 [15].

Svp is exclusively expressed in R3/R4 and R1/R6 pairs of the outer PRs in early retina development. Initially, Sal is expressed in the R3/R4 PRs in order to promote Svp expression. Later, Svp represses Sal in R3/R4 PRs in order to prevent the transformation of R3/R4 into R7 [16]. Similarly in larval PRs Svp is repressing Sal in secondary precursors [7].

Intriguingly, in R8 development in the adult retina Sens also provides two temporally separable functions: First, during R8 specification, lack of Sens in precursors results in a transformation of the cell into R2/R5 fate [26]; second, during differentiation, Sens counteracts Pros to inhibit R7 cell fate and promotes R8 cell fate [25], [33], [34]. Thus, Sens is an early genetic switch in R8-PRs and larval Rh5-PRs that represses an alternate cell fate.

The lack of Sens results in a larval eye composed of only Rh6-PRs. Thus, the default state for both primary and secondary precursors is to differentiate into Rh6-expressing PRs. Rh6 is also the default state in adult R8 PRs: In the absence of R7 PRs (e.g. *sevenless* mutants) that send a signal to a subset of underlying R8 PRs, the majority of R8 PRs express Rh6 [12], [35]. Thus, the genetic pathway initiated by the Sens pulse ensures that primary precursors choose a distinct developmental pathway by repressing the Rh6 ground state. The mechanisms that initiate and control this pulse of Sens remain to be discovered.

In larval PRs as well as in the formation of sensory organ precursors (SOP) in the wing, Sens functions as a binary switch between two alternative cell fates. In the larval eye, this switch occurs when Sens is expressed in one cell type and not in the other. However, during wing disc development the cell fate choice in SOP formation is controlled by the levels, and not the presence or absence of Sens expression: high levels of Sens act synergistically with proneural genes to promote a neuronal fate, while in neighboring cells, low levels of Sens repress proneural gene expression, thereby promoting a non-SOP fate [36]. Thus, Sens uses distinct molecular mechanisms to act as a switch between Rh5 versus Rh6-PR cell fate and SOP versus non-SOP cell fate.

### Sens mediates a survival signal in many developmental contexts

Transcription factors regulate developmental programs in a context- dependent fashion [34]. An example is Sens, which has distinct functions in BO and eyelet development. First, during embryonic development, Sens acts as a key cell fate determinant by regulating transcription factors controlling PR-subtype specification. Second, during metamorphosis Sens inhibits ecdysone-induced apoptotic cell death. Third, in the adult eyelet Sens promotes Rh6 expression. Interestingly, the pro-survival function of Sens appears to be a conserved feature of Sens in other tissues and also in other animal species. In the salivary gland of *Drosophila*, Sens acts also as a survival factor of the salivary gland cells under the control of the bHLH transcription factor Sage [37]. *pag-3*, a *C.elegans* homolog of Sens is involved in touch neuron gene expression and coordinated movement [38], [39]. Pag-3 was shown to act as a cell-survival factor in the ventral nerve cord and involved in the neuroblast cell fate and may affect neuronal differentiation of certain interneurons and motorneurons [40]. In mice, *Gfi1* is expressed in many neuronal precursors and differentiating neurons during embryonic development and is required for proper differentiation and maintenance of inner ear hair cells. *Gfi1* mutant mice lose all cochlear hair cells through apoptosis, suggesting that its loss causes programmed cell death [41]. Taken together, these findings support that Sens and its orthologs function in cell fate determination and cell differentiation both in nervous system formation, but also play an essential role in the suppression of apoptosis.

### Hazy is critical for larval, adult and eyelet PR development

Hazy plays distinct roles in larval PRs and during metamorphosis. First, Hazy is essential during embryogenesis for proper PR differentiation. This early function of Hazy is essential for PRs to differentiate properly during embryogenesis, to express Rhodopsins and to subsequently maintain Rhodopsin expression during larval stages. This function of Hazy is similar to its role in rhabdomere formation in adult PRs and subsequent promotion of Rh6 expression, although it is not required for Rh5 in the adult retina [29]. It is likely that Hazy exerts this function by binding to the RCSI site of the *rhodopsin* promoters, as has been suggested for the adult retina [31]. Second, during metamorphosis Hazy is required in Rh6-PRs to repress *sens*, thus allowing these cells to undergo apoptosis. This highlights the reuse of a small number of TFs for distinct functions in the same cell type at distinct time points of PR development. How these temporally distinct developmental programs are controlled on a molecular level remains unresolved. It seems likely that the competence of the cell to respond to a specific transcription factor changes during development.

### Comparison between gene regulatory networks specifying the same Rhodopsin fate in larval and adult PRs

*rh5* and *rh6* are expressed in different PRs at different developmental stages: *rh5* is expressed in the larval eye and in the adult retina, whereas *rh6* is expressed in the larval eye, the adult eyelet and the adult retina. However, the gene regulatory networks controlling *rhodopsin* expression are distinct in these organs. In the adult retina, a bistable feedback loop of the growth regulator *melted* and the tumor suppressor *warts* acts to specify Rh5 versus Rh6 cell fate, respectively [11], while in the larva, Sens, Sal, Svp and Otd control Rh5 versus Rh6 identity [7] whereas Hazy has been shown to maintain Rhodopsin expression. A third genetic program acts downstream of EcR during metamorphosis in Rh5-PRs to switch to Rh6, which requires Sens.

An intriguing question is how the developmental pathways to specify Rh5- or Rh6-cell fates converge on the regulatory sequences of these two genes. It seems likely that parts of the regulatory machinery acting on the *rh5* and *rh6* promoters are shared between the larval eye, adult retina and eyelet, especially as short minimal promoters are functional in all three different contexts (Rister, Tsachaki and Sprecher, unpublished). Future experiments will show how the activity of the identified trans-acting factors is integrated on these promoters to yield context-specific outcomes.

## Materials and Methods

### *Drosophila* strains and genetics

Wild-type Canton S or the Sp/CyO; TM2/TM6b strains were used as controls in all cases. The following fly strains used were *sal^16^* [42], *svp^E22^* [43], *otd^uvi^* [18], *sens^E2^* and UAS-*sens* [44], *Pph13* ^hazy^ (here termed hazy), heat shock-*Hazy* [27], UAS-*EGFR^DN^* [45], UAS-*H2B::YFP* (anti-GFP antibody/biogenesis recognizes the YFP antigen), UAS-*ato*, UAS-*p35*, UAS-*EcR^DN^* (isoform B2), UAS-*mCD8::GFP*, *GMR*-Gal4 and *rh5*-Gal4 (Bloomington Stock Center), UAS-*sens^RNAi^* (VDRC Stock Center), and *so*-Gal4 [46]. *sens^E2^*, *svp^E22^* double mutants were generated by recombination.. All the crosses were grown at 25°C except RNAi experiments, which were performed at 29°C. For analysis of 3×P3-RFP [30] expression, we used the 3×P3-RFP marked attB integration site at 86Fb [47].

### Generation of transgenic flies

*Chp^4.5^*-Gal4 flies were made by amplification of 4.5 kb of sequence upstream of the *chaoptin* gene from genomic DNA using primer pairs AC25/AC27 and the GeneXL PCR amplification system and introducing a NotI restriction site to the 5′ end and a BglII site to the 3′ end. Amplified fragments were cloned into the NotI/BamHI site of a Gal4 vector containing a hs43 promoter (hs43-Gal4) [48]. The primers were:

AC25 TGAC**GCGGCCGC**GTCGACGAGTCTTTATGC NotI

AC27 TGAC**AGATCT**CGATCGAACATGGAGGCGCGA BglII

The cDNA of *hazy* was subcloned into the pUAST/attB vector [47] between the BglII and NotI sites. The pCDNA3 plasmid containing the cDNA of *hazy* was kindly provided by A. Zelhof (Indiana University, Bloomington).

The *rh6* (−227/+121) and *rh5* (−256/+50) minimal promoters were generated using the following primers flanked with 5′BglII and 3′ NotI sites for directional cloning into a transformation plasmid containing *eGFP*, a *miniwhite* marker and an *attB* site:

*rh5* fw: **AGATCT** AACATGTAAAGCTTGTAAAA

*rh5* rev: **GCGGCCGC** TAGTTTCCTTTGCAGGTCGAC

*rh6* fw: **AGATCT** GGGTGGGTGGTACCTCAAAC

*rh6* rev: **GCGGCCGC** GGTGGCGCTTCGGTGGTGGCTTC

RCSI deletions were generated using the Stratagene QuikChange site-directed mutagenesis kit with the following primers:

DRCSRh6A: TGGATTGGCCAAGTGCCGGCGGGCAATTAGTCTAAGACG

DRCSRh6B: CGTCTTAGACTAATTGCCCGCCGGCACTTGGCCAATCCA

DRCSRh5A: AATGGTCACCACTTAATCCGTCTTTTGGCGGGCTATAAAAGCAT

DRCSRh5B: TTACCAGTGGTGAATTAGGCAGAAAACCGCCCGATATTTTCGTA

The UAS-*hazy* construct, as well as *rh6-GFP*, *rh5-GFP, rh5-GFP^ΔRCSI^* and *rh6-GFP^ΔRCSI^* reporters were all inserted into the 86Fb site on the third chromosome using the φC31 site-specific integration system [47].

### Immunohistochemistry

Embryos were dechorionated, fixed and immunostained according to a previously described protocol [49]. Dissection and immunostaining of the larval eye and the eyelet have been described previously [7], [21]. The samples were mounted in Vectashield H-1000 (Vector laboratories). Primary antibodies and dilutions were as follows: rat anti-Elav 1∶30 and mouse anti-FasII 1∶30 (Developmental studies Hybridoma bank), rabbit anti-Sal 1∶300 [42], mouse anti-Svp 1∶100 [50], rabbit anti-Hazy 1∶500 [27], sheep anti-GFP 1∶1000 (Invitrogen), guinea pig anti-Sens 1∶800 [44], rat anti-Kr 1∶200 [51], rabbit anti-Rh6 1∶10000 [52], mouse anti-Rh5 1∶20 [53] and rabbit anti-Otd 1∶200 [54]. The secondary antibodies were anti-rabbit, anti-mouse, anti-rat conjugated with Alexa-488, Alexa-555 or Alexa-647, anti-guinea pig DyLight 549 and anti-Sheep DyLight 488 (Jackson Immunoresearch). All secondary antibodies were developed in donkey and/or goat and used in 1∶200 dilution.

### Laser confocal microscopy and image processing

The confocal microscope for the analysis of the samples was a Leica TCS. The picture size was 512×512 or 1024×1024 pixels and the optical sections ranged from 0.8–1.5 µm depending on the sample. The images acquired were post-processed using the Fiji software and Adobe Photoshop CS3.

## Supporting Information

Figure S1 Sens and Hazy expression in *EGFR^DN^* and *ato* overexpression. (A, B) Sens expression (red) in wild-type and in *so*-Gal4/UAS-*EGFR^DN^* stage 12 embryonic PRs, stained against FasII (Green); single confocal sections. Sens expression was not affected in *so*-Gal4/UAS-*EGFR^DN^* embryonic PRs. (C, D) Sens (Red) and Hazy (Blue) expression in wild-type and in *so*-Gal4/UAS-*EGFR^DN^* stage 15 embryonic PRs, stained against FasII (Green); single confocal sections. Larval eye precursors consist of 4 cells (marked by Hazy expression) and no change of Sens and Hazy expression was found in *so*-Gal4/UAS-*EGFR^DN^* embryonic PRs. (E, F) Sens (Red) expression in wild-type and in *so*-Gal4/UAS-*ato* stage 12 embryonic PRs, stained against FasII (Green); single confocal sections. Sens expression was not changed in *ato* overexpression in the embryonic PRs.
(TIF)

Figure S2 *chp^4.5^*-Gal4 expression during different developmental stages. (A) GFP expression (green) in the embryonic PRs in *chp^4.5^*-Gal4/UAS-*mCD8*::GFP at stage 16, stained with FasII (red) and Elav (blue); single confocal section. No GFP expression was found in the embryonic PRs. (B, B′ C, C′) GFP expression (green) in the larval eyes of *chp^4.5^*-Gal4/UAS-*mCD8*::GFP larval first and third instar, stained with Chp (red) and Elav (blue). GFP expression was found in all PRs in the larval eye. (D, D′) GFP expression in the eyelet of *chp^4.5^*-Gal4/UAS-*H2B*::YFP adult animals, stained with Rh6 (red) and Elav (blue). All the cells in the eyelet expressed GFP.
(TIF)
