# Supplementary figures and images for: Binary Cell Fate Decisions and Fate Transformation in the Drosophila Larval Eye
Source: PLoS Genet. 2013 Dec 26;9(12):e1004027. doi: 10.1371/journal.pgen.1004027 (PMC3873242; doi:10.1371/journal.pgen.1004027)

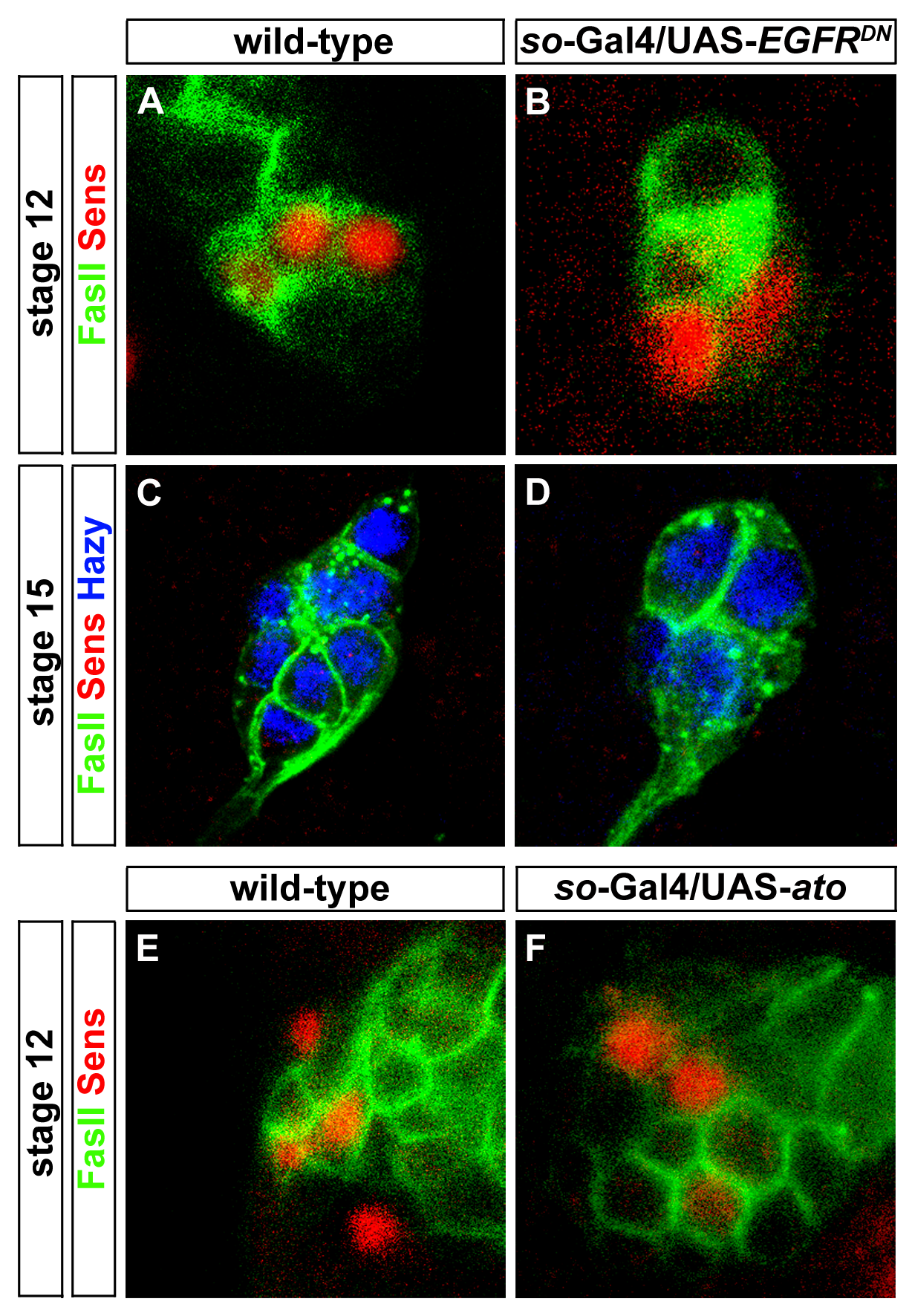

Supplement: Figure S1 — Sens and Hazy expression in EGFRDN and ato overexpression. (A, B) Sens expression (red) in wild-type and in so-Gal4/UAS-EGFRDN stage 12 embryonic PRs, stained against FasII (Green); single confocal sections. Sens expression was not affected in so-Gal4/UAS-EGFRDN embryonic PRs. (C, D) Sens (Red) and Hazy (Blue) expression in wild-type and in so-Gal4/UAS-EGFRDN stage 15 embryonic PRs, stained against FasII (Green); single confocal sections. Larval eye precursors consist of 4 cells (marked by Hazy expression) and no change of Sens and Hazy expression was found in so-Gal4/UAS-EGFRDN embryonic PRs. (E, F) Sens (Red) expression in wild-type and in so-Gal4/UAS-ato stage 12 embryonic PRs, stained against FasII (Green); single confocal sections. Sens expression was not changed in ato overexpression in the embryonic PRs. (TIF) [file pgen.1004027.s001.tif]

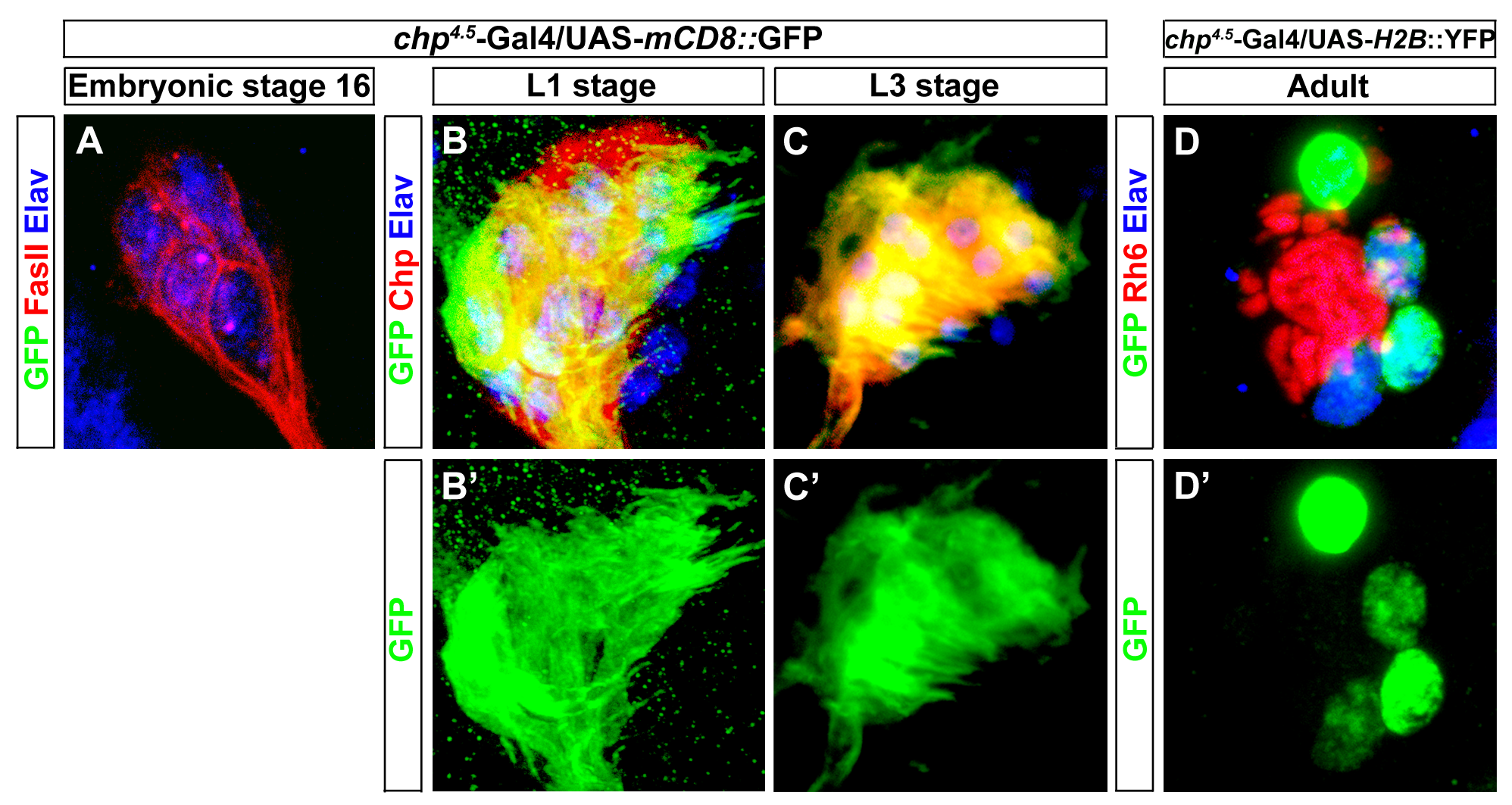

Supplement: Figure S2 — chp4.5-Gal4 expression during different developmental stages. (A) GFP expression (green) in the embryonic PRs in chp4.5-Gal4/UAS-mCD8::GFP at stage 16, stained with FasII (red) and Elav (blue); single confocal section. No GFP expression was found in the embryonic PRs. (B, B′ C, C′) GFP expression (green) in the larval eyes of chp4.5-Gal4/UAS-mCD8::GFP larval first and third instar, stained with Chp (red) and Elav (blue). GFP expression was found in all PRs in the larval eye. (D, D′) GFP expression in the eyelet of chp4.5-Gal4/UAS-H2B::YFP adult animals, stained with Rh6 (red) and Elav (blue). All the cells in the eyelet expressed GFP. (TIF) [file pgen.1004027.s002.tif]
